# Husbandry Conditions and Welfare Outcomes in Organic Egg Production in Eight European Countries

**DOI:** 10.3390/ani10112102

**Published:** 2020-11-12

**Authors:** Lisa Jung, Christine Brenninkmeyer, Knut Niebuhr, Monique Bestman, Frank A. M. Tuyttens, Stefan Gunnarsson, Jan Tind Sørensen, Paolo Ferrari, Ute Knierim

**Affiliations:** 1 Farm Animal Behaviour and Husbandry Section, University of Kassel, Nordbahnhofstraße 1a, 37213 Witzenhausen, Germany; c.brenninkmeyer@naturland.de (C.B.); uknierim@uni-kassel.de (U.K.); 2 Institute of Animal Husbandry and Animal Welfare, University of Veterinary Medicine, 1210 Vienna, Austria; vmu-tierhaltung@vetmeduni.ac.at; 3 Department of Agriculture, Louis Bolk Institute, Kosterijland 3–5, 3981 AJ Bunnik, The Netherlands; m.bestman@louisbolk.nl; 4 Farm Animal Welfare and Behaviour Group, Animal Sciences Unit, Institute for Agricultural and Fisheries Research (ILVO), Scheldeweg 68, B-9090 Melle, Belgium; frank.tuyttens@ilvo.vlaanderen.be; 5 Department of Nutrition, Genetics and Ethology, Faculty of Veterinary Medicine, Ghent University, Heidestraat 19, B-9820 Merelbeke, Belgium; 6 Department of Animal Environment and Health, Swedish University of Agricultural Sciences (SLU), P.O. Box 234, S-532 23 Skara, Sweden; stefan.gunnarsson@slu.se; 7 Department of Animal Science, Aarhus University, Blichers Allé 20, DK-8830 Tjele, Denmark; jantind.sorensen@anis.au.dk; 8 CRPA, Research Centre for Animal Producton, V.le Timavo 43/2, 42121 Reggio Emilia, Italy; p.ferrari@crpa.it

**Keywords:** animal welfare, laying hen health, feather pecking, keel bone damage, laying hen, parasite load, free-range use

## Abstract

**Simple Summary:**

An important challenge for the further development of organic egg production is to reach and maintain a high level of animal welfare. In a European research project including eight countries, the welfare state in 107 laying hen flocks as well as their housing and management were recorded during two farm visits at peak and end of lay. Data analysis aimed to reveal factors that may help to prevent or reduce welfare problems. Large variation between flocks indicated options for improvement with regard to mortality, feather and injurious pecking, parasitic infestation and keel bone damage. Results of the project indicate that (i) decreasing mite and worm infestation and (ii) providing an attractive covered veranda help to decrease mortality; (iii) maximising access to the free-range to decrease injurious pecking and *Ascaridia galli* infection; (iv) feeding sufficient protein levels and (v) providing adequate litter is preventive against feather pecking and cannibalism; (vi) ensuring that the birds have sufficient weight and (vii) preventing accidents by adequate hen house facilities and light conditions contributes to reduced keel bone damage. These primarily management-based measures have the potential to improve bird welfare both in terms of behavioural and health aspects in organic egg production systems.

**Abstract:**

In the European research project HealthyHens, welfare indicators as well as husbandry and management conditions were recorded in 107 organic laying hen farms in eight countries. Farms were visited at peak and end of lay. Egg production was on average comparable to breeder specifications. A mean mortality of 5.7% and mean prevalences of footpad lesions of 30.5%, keel bone damage of 44.5%, 57.3% of flocks with on average >200 Ascarid eggs per gram faeces and 28.2% of flocks with >100 mites/trap were recorded. A large variation between flocks indicated options for improvement. Based on the results, the following measures can be recommended: (i) decreasing mite and worm infestation and (ii) providing an attractive covered veranda, because of their association with decreased mortality; (iii) maximising access to the free range, because of its relation to decreased *A. galli* infection and less injurious pecking; (iv) feeding sufficient protein levels and (v) providing adequate litter as preventive measure against feather pecking and cannibalism; (vi) ensuring that the birds have sufficient weight and (vii) preventing accidents by adequate hen house facilities and light conditions to reduce keel bone damage. These primarily management-based measures have the potential to improve bird welfare both in terms of behavioural and health aspects.

## 1. Introduction

There is a growing public concern for animal welfare [1] in conventional intensive farming practice, which has led to an increased popularity of animal production systems that offer outdoor access, including organic egg production systems [2,3]. Consumers expect that free-range access enhances welfare compared with hens housed indoors [4]. Organic egg production generally accounts for a relatively low proportion of overall egg production but has substantially increased in recent years and gained considerable significance in some EU member states. For example, in Denmark laying hens kept under organic production rules made up more than 31% of the total production, in Sweden more than 16% and in Austria and Germany around 12% in 2018 (calculated from [5]). Besides limited group sizes of 3000 hens and stocking densities of not more than six hens/m^2^, a free-range area is mandatory in organic egg production. The free range can contribute to improved welfare of poultry, but can also increase risks for infectious diseases [6,7] and mortality due to predators [8,9]. On the positive side, for instance, associations between the increased use of an outdoor area and lower levels of feather pecking have been found (reviewed by [10]).

An important challenge for the further development of organic egg production is to reach and maintain a high level of animal welfare. There are a number of welfare aspects which constantly need attention (e.g., [11,12]), such as feather pecking (FP), worm infestation, keel bone damage (KBD), foot pad lesions (FPL) or mortality rates. For instance, in the past, moderate to severe feather damage has been reported in 71% of organic flocks [13], with on average 50% of birds being affected [14,15]. Dutch data from about seven years later indicate an improvement with 32% of farms having feather damage problems [16]. Although a considerable amount of work has already been done on feather pecking (e.g., reviewed by [10,17]), it is still a major welfare threat in all housing systems. The main causes of feather pecking may vary over the years; some risk factors (for example in the rearing period) may be successfully addressed, while others may newly appear due to changing circumstances (for example 100% organic feed). The prohibition of routine beak trimming in organic layer hens (which is still a predominant practice in the conventional production of many countries) may increase the risk of injurious pecking in organic flocks. Feeding management, in particular, determines to what extent hens are offered the nutrients they need and how much time they are occupied with functional pecking behaviour. From 2021 onwards, the EU regulation [18] will require organic laying hen farmers to use rations based on 100% organic feed ingredients. Suboptimal diets may be a risk factor for feather pecking as well as cannibalism. Cannibalism (excluding vent pecking) was reported by farmers in one study at 22.6% of partly repeated visits in 119 barn, free-range or organic flocks [19], while Finnish organic farmers in the study of Kaukonen and Valros [20] found signs of cannibalism in 0.5% (range 0–7%) of their flocks. However, cannibalism is a serious welfare problem in poultry that causes pain and often leads to the victim’s death, beside economic losses.

Endoparasite infection is widespread in organic poultry production. *Heterakis gallinarum* were found in 89% and *Ascaridia galli* in 84% of free-range flocks, including organic farms, in a British study [7]. Jansson et al. [21] reported a prevalence of 77% of *A. galli* and *H. gallinarum* combined for free-range and organic farms in Sweden. Consequences of *A. galli* infections in laying hens include decreased locomotive activity in combination with increased feeding, indicating a higher nutritional need [22]. The caecal worm *H. gallinarum* seldom causes clinical problems but may serve as the intermediate host for the protozoan parasite *Histomonas meleagridis*. This in turn causes Histomoniasis, a form of blackhead disease, which can lead to very high flock mortality, as there is a lack of allopathic treatment possibilities [23].

A further increasing welfare concern in non-cage systems are fractures and deviations of the keel bone. Reported prevalences of keel bone damage during lay range from 3 to over 90% affected hens/flock [24,25,26]. In organic flocks, Bestman and Wagenaar [16] found a mean of about 21%, Grafl et al. [27] of 25% of the hens with damaged keel bones.

Another common problem in laying hens housed in non-cage systems are foot pad lesions (FPL), namely dermatitis and bumble foot. In addition, hyperkeratosis is a rather frequent finding, but which is comparatively less prevalent in birds with free-range access (33.5 vs. 46.7% of birds, [25]), and the impact on welfare is lower compared to lesions and swellings. Reported FPL prevalences range from on average 13% of hens per flock, with 78% of 49 Dutch organic layer flocks being affected [16], over 28.8% in 47 Belgian aviaries [25] to 40% in Austrian non-cage systems [28]. Grafl et al. [27] assessed FPL at the slaughter line in seven Austrian organic flocks that were all affected with on average 52.4% of hens per flock.

Fractures of the keel bone [29,30] as well as FPL are most likely painful for the hens. Therefore, these multifactorial health problems impair animal welfare and show that aspects of housing and management are not well adapted to the birds’ physiology and behaviour.

Mortality in layers is affected by many factors which often relate to various other animal welfare problems. Thus, mortality is regarded an iceberg indicator, accumulating information on these other welfare problems [31,32]. Concerning mortality, von Borell and Sørensen [33] cited Danish results, finding an average mortality rate in organic flocks of 17%. Leenstra et al. [34] reported higher mortality rates for organic compared to conventional free-range flocks, with a mean of 12.0% mortality until 60 weeks of age (WoA) for 57 organic flocks in the Netherlands. Later, Leenstra et al. [35] reported a mean mortality of 7.9% in 42 organic Dutch flocks, which may indicate that hen health improved over time. Likewise, Steenfeldt and Nielsen [36] reported lower mortality rates of 3.0–3.4% in two investigated organic flocks.

For some poultry welfare problems, fundamental knowledge is available from experimental investigations. For a better understanding of the multifactorial nature of these problems on farm, the number of epidemiological studies has increased in recent years. However, the number of epidemiological studies concerning welfare in organic layer farms is very limited (overview by [31]). Therefore, information regarding the welfare or health status as well as regarding influencing factors under European organic conditions is lacking.

This paper reports from the European research project HealthyHens (http://orgprints.org/20715/), which addressed the issues discussed above. It presents an overview of relevant outcomes regarding organic laying hen welfare under varying production and husbandry conditions in eight European countries. It further investigates the potential contributions of behaviour, health and performance aspects to mortality rates, and based on additional earlier analyses of the data regarding parasite infestation [37], feather and injurious pecking [38] as well as keel bone damage [39] proposes general recommendations for organic egg production.

## 2. Materials and Methods

This study was carried out in compliance with the national animal welfare laws. For the observations and clinical scoring, the competent welfare authorities required no formal approval procedure. Data were recorded under unchanged farm conditions. The participation of farms in the study was voluntary, and the farmers were informed about the purpose and methods of the study by written project information in advance. They were assured that all information would be treated anonymously, and that they could withdraw from the study at any time.

Farm selection aimed at reflecting the main commercial organic laying hen farming conditions in each of the eight participating countries. Thus, conditions which represented only a small minority of organic laying hen places and which were expected to be too rare for statistical analysis were excluded. This applied to farms with mobile hen housing relocated frequently during the laying period and farms with less than 500 hen places. In the period February 2012 to March 2014, 114 farms had been visited. Records from seven flocks were omitted due to inconsistency of data; therefore, the cross-sectional study included 107 organic layer flocks (in total 259,155 hens) across eight European countries: Austria (AT: 25 flocks), Germany (DE: 19), Denmark (DK: 15), Italy (IT: 15), United Kingdom (UK: 10), Sweden (SE: 9), Netherlands (NL: 7) and Belgium (BE: 7). Farm size ranged from 500 to 3000 hen places (33.0% of the sample), 3001 to 5000 (12.5%), 5001 to 10,000 (23.9%), 10,001 to 30,000 (25.0%) to more than 30,000 hen places (5.7%). Hen places per farm were defined as the capacity of all hen houses, managed by the same person, in terms of the number of hens that can be kept at a time, and this could comprise hen houses at different locations. For practical reasons, farms purchasing compound feed were prioritised in order to be able to use feed declarations as an information source.

Data collection took place at peak of lay (mean of 35.9 WoA), range 29–47 WoA) and at a second time towards the end of lay (mean of 62.4 WoA, range 52–73 WoA). To ensure statistical independence of flocks, data were recorded from only one flock per farm.

Management data were collected in an interview with the farm manager or the person responsible for hen care using a standardised questionnaire. Data on housing conditions in the hen house, the covered veranda and free range, including the structural and functional elements (e.g., feeders, perches), were directly recorded. Use of the free range was assessed during each visit at three standardised times with regard to the time of sunset. The number of birds on the range was counted in three different zones and, if present, on the covered veranda. The percentage of hens using the covered veranda or the free range was defined as the maximum percentage of hens counted in the respective area at a time.

The assessment of endoparasite burden was performed by counting eggs and Coccidia oocyst in faeces (McMaster technique). At all farm visits, 14 to 21 fresh intestinal droppings were collected from the floor of the housing facilities, and faecal egg counts were performed for these individual droppings to determine egg and oocyst numbers per gram faeces (EPGs) as described in Thapa et al. [37]. As visual differentiation between *Ascaridia galli* and *Heterakis spp.* eggs is doubtful with McMaster technique, eggs of both species were counted jointly as Ascarid eggs.

Burden of red mites (*Dermanyssus gallinae*) was screened using 10 card board mite traps per flock each at either the summer visit (all farms) or both visits (58 farms). The traps were fixed on the underside of the cross supports carrying the perches, or the perches next to the cross supports in the evening and left in place for 7 days. After removing the traps in the morning, they were transferred individually into zip-locker plastic bags and placed in a freezer at −20 °C for at least 24 h. Each sample was tapped out and distributed evenly in a petri-dish with a grid. The grid served to estimate the number of mites by counting the number of mites within one square and multiplying this by the number of occupied squares. Based on this number, a score from 0 to 5 was assigned (0 = no mites, 1 = 1 to 10 mites, 2 = 11 to 100, 3 = 101 to 1000, 4 = 1001 to 10,000 and 5 = more than 10,000).

During the second visit which was on about half of the farms in “summer” (April to September) and on the others in “winter” (October to March), 50 hens per flock were assessed concerning plumage condition and wounds based on the LayWel scoring scheme [40]. For the same 50 hens, keel bone damage and foot pad lesions were evaluated (Table 1) as well as beak status (trimmed or non-trimmed). Furthermore, the same 50 hens were weighed to calculate the proportion of underweight hens, defined as hens being 11% or more below breeder recommendations.

Prior to the start of farm visits, 12 assessors were trained at a joint training occasion in order to achieve a consistent scoring, which was tested as inter-assessor agreement on-farm for all measures that required scoring. Prevalence-adjusted bias-adjusted kappa values (PABAK) [41] were calculated for each observer pair and each parameter. PABAK values ≥ 0.4 were considered acceptable based on the classification of Fleiss et al. [42]. In one case, scoring deviated substantially, and a subsequent round of trainings and testing using photos was necessary to achieve acceptable assessor agreement before data collection started. The PABAK values regarding the assessment of health indicators are listed in Table 1. Two rounds of inter-laboratory tests were performed on dropping samples standardised for different Ascarid egg and Coccidia oocyst counts per gram faeces, using regression lines evaluated visually in comparison to the Danish laboratory as a reference, in order to reach satisfactory agreement.

Beside descriptive statistics concerning housing and management practices as well as animal scores, a multiple linear regression was calculated with forward stepwise selection for analysing possible influencing factors on bird mortality. Included factors were the performance and health outcomes as well as behavioural measures (percentage of hens using the free range; percentage of hens using the covered veranda, with 0% set when no veranda was present). In addition, a variable was created to summarise the overall health problems of a flock. It included the percentage of health problems (dichotomised into present or not, according to Table 2) from all possible problems given in Table 2. Model diagnostics were carried out graphically, for evaluating linearity of variables and homogeneity of variance by scatter plots of studentised residuals and unstandardised predicted values. Normal distribution of residuals was evaluated with histograms and p–p plot. The absence of strong collinearity between factors was checked using collinearity statistics and variance inflation factor, where values should not be >5 [43]. The absence of influential data points was checked by Cooks’ distance ≤ 1.0 [44]. All statistics were carried out using the software SPSS (version 24).

## 3. Results

An overview of the various European housing and management conditions is presented in Table 3 and Table 4.

### 3.1. Free Range Properties and Management

Available free-range area varied from ≤4 m^2^/hen (fulfilling EU organic minimum requirements or less) on 19% of the farms to >16 m^2^/hen on 7% of the farms. The highest proportion (41%) comprised farms providing 4 to 8 m^2^/hen. On most farms (61%), access to the range was given depending on the weather conditions, but definitions of unsuitable weather varied. The most common reason for keeping hens inside was snow (38%), but rain in general (21%) or heavy rain (12%) were also common reasons for keeping the pop-holes closed. A total of 16% of the flocks were kept inside if the temperature dropped below a certain value, which ranged from −15 to 1 °C (mean −1.6, median 0 °C). In certain areas, snow and temperature considerations led to no outside access in winter. In some cases (5%) pop-holes were irregularly closed for other reasons than weather, e.g., predators or pandemic situations, sometimes not specified by the farmers. A total of 34% of the farms opened the pop-holes daily, independent of weather and other reasons. Range rotation within batches was performed on 13% of the farms, and 15% rotated ranges between batches. On 20% of the farms, the soil in front of the hen house was replaced or turned regularly, while 14% replaced or turned it irregularly and 66% never.

### 3.2. Housing Equipment

The flooring of elevated surfaces consisted of plastic grid on 51% of the farms, while 30% had metal grids, 5% wire mesh and 14% other materials. Perches were most commonly made of metal (70%) and wood (54%) and less often plastic (17%), whereby multiple materials per farm were possible. Incandescent light was used in about 42% of the hen houses, LEDs in 15%. The fluorescent lights used were low frequency lights in 15% of houses (however, in 22%, the frequency was not identified, and multiple light systems were also possible). In 92% of the farms, the nests had ‘nest curtains’ (plastic flaps) partly covering the entrance, while in 2% curtains completely covered the entrance and in 6% of farms nests were without curtains.

### 3.3. Feeding

In 97 out of 107 farms the farmers made use of the derogation that allows to supplement rations by a limited amount of non-organic protein feed components. Feed consisted of medium ground mash on 53% of farms, 10% of the farms fed course ground and 5% fine ground mash. Crumbled pellets were found on 21% and complete pellets on 11% of the farms. The number of feed phases varied largely: 24% of the flocks received one single phase during the entire laying cycle, 31% two phases, 29% three phases, 12% four phases and 1% five or more. Protein content in the feed rations at week 55 of the hens’ life varied between 14.6 and 22.2% with energy contents between 10.2 and 12.8 MJ ME/kg dry matter and crude fibre contents from 3.0 to 8.5%.

### 3.4. Health Indicators and Production Data

The distribution of welfare and production data, collected around the peak and around the end of lay, is presented in Table 5. Mean laying performance per hen day varied widely between flocks at the peak of lay (26 percentage points: from 73 to 99%) and even more towards the end of lay (36 percentage points). Prevalences of most hen welfare problems at the end of lay (mean 62 WoA) covered the complete or nearly complete scale (80 to 100 percentage points). The percentage of the various health problems on flock level, as described in Table 2, ranged from 12.5 to 100% (mean: 62.3%, median: 62.5%).

The significant final model (F (3,52) = 12.030, *p* < 0.001, *n* = 56) explained about 34% of the variation of mortality rates between flocks and contained three factors. A lower laying performance at peak of lay, a higher mite burden and fewer hens using the covered veranda were associated with higher mortality rates (Table 6).

## 4. Discussion

The farms included in the study either complied solely with EU organic standards, or additionally with standards of organic producer associations. Even though the study was aimed at reflecting the main commercial organic laying hen farming conditions in each of the eight participating countries, a representative spatial distribution of farms within countries was not always feasible and, finally, the willingness of the farmers to participate in the study was a precondition for selection. Nevertheless, the sample is expected to cover the major production conditions of organic eggs produced in the participating countries.

While brown layers were the predominant hybrid in most countries, in SE all and in DK the majority (83%) of the recruited flocks consisted of white layers.

EU Regulation [18] requires that mutilations such as beak trimming are not carried out routinely in organic farming. In total, hens from 14 flocks in three countries (13.1% of all assessed flocks) had undergone beak trimming to differing degrees, including infrared treatment with removal of a smaller part of the beak. It is unclear whether on these farms beak trimmed hens were used routinely. A lack of availability of chicks with intact beaks could be a reason for beak-trimmed hens in the sampled flocks in the three countries, BE, IT and the UK. These countries had the lowest proportion of laying hens kept under organic husbandry conditions of the eight included countries (2.0 to 2.6% in 2014, calculated from data of EU Commission [45]). Consequently, hatcheries might not be prepared for this market and beak trim all chicks shortly after hatch. On the other hand, in all three countries, flocks were found with non-beak trimmed hens. Possibly communication about organic requirements should be intensified and an earlier planning of new flocks could help to solve logistical problems.

In six flocks, rations of 100% organic feed were provided, which will be mandatory from 2021 onwards [18] for laying hens. This result indicates the challenge to reach 100% organic food rations while ensuring the nutritional requirements of the birds. To use 100% organic products is highly important for the organic sector, but an optimal nutrition is one of the most important influencing factors concerning bird health and has to be ensured as well. Therefore, new strategies that safeguard sustainable and well-balanced feeds for organic laying hens are urgently needed [46].

The proportion of flocks feeding pelleted feeds was surprisingly high (32%) considering the increased risk of feather pecking related to pelleted feed for laying hens (reviewed by [10,47]). However, pelleted feed has the advantage to better safeguard uptake of all offered nutrients. Bestman et al. [38] in the current data set found low dietary protein contents to be associated with increased feather damage in brown layers. Problems may be aggravated in mash by insufficient uptake of the fine particles of the protein feed. In an otherwise stimulating environment, this may outweigh the potential negative effects of decreased feeding times of the pelleted feed.

In several aspects, legal provisions and the interpretation of the relevant EU legislation regarding husbandry conditions for organic laying hens differ between the participating countries. Additionally, in most countries stricter or more detailed private standards of organic producer or marketing associations exist, leading to different practices even within countries. Concerning multi-tier systems, for instance, for all countries the EC Directive for the protection of laying hens [48] limits the possible number of tiers to three additional to the floor. However, in DK the upper limit is set at two, and in AT at two plus perches. In The Netherlands it is not allowed to keep more than nine hens per floor m^2^, which in practice works as a limit to the number of tiers. The Soil Association in the UK only allows single-tier housing of the hens. In line with this, no multi-tier flock could be recruited in the UK and the highest proportions of multi-tier systems have been found in DE, SE and AT. All but two multi-tier flocks had access to a covered veranda which might compensate the higher stocking densities per floor m^2^ in the hen house. Indoor stocking densities exceeded legal requirements on about 6% of the participating farms. However, on the majority of farms, more floor space than the legal minimum requirement was offered which might reflect experiences that this helps to mitigate problems. A number of practice recommendations list low stocking density as a preventive measure concerning feather pecking, although only very few scientific studies confirm this association (reviewed by [10]). The proportion of ground surface covered with litter material exceeded minimum requirements in all hen houses but one. On one farm, on the other hand, the complete ground surface of the barn system was littered, which does not fulfil the organic requirements that a “sufficiently large part of the floor area available to the hens must be available for the collection of bird droppings” [18]. Almost one third of the participating farms did not add any litter material during the production cycle, but only between batches. This practice is critical, as based on the present data set, Bestman et al. [38] showed that not providing new litter material during the laying cycle was correlated with higher prevalences of pecking wounds, at least in white hens. This is in line with other findings that poor litter availability or quality increase the risk for feather pecking (reviewed by [10]), in addition to increasing the risk for foot pad lesions if the litter is wet [49].

In the light of national certification rules, it seems plausible that nearly all hen houses were equipped with covered verandas in SE, DE, NL and AT. In DE and in SE for hen houses built or put into operation after a certain date (DE: August 2006; SE: 2012), covered verandas are mandatory for hen houses with access to free-range. NL and IT are affected by rules applied in Germany, as organic eggs are produced partly for the German market and the German certification body KAT demands the presence of a covered veranda for imported organic eggs. In DK, DE, NL and SE, the ground surface of the covered veranda is considered as available surface if it is continuously accessible. In AT it is partly considered, whereas in the UK it is not considered, even though a median access time to the veranda of 24 h was also found in the UK, and not only in DE, DK and SE. Projecting roofs were very rare (11%) and were an addition rather than an alternative to the covered veranda in most cases.

The number of hen house buildings per site was the highest in the UK, and, at the same time, group size and flock size medians were lowest. This reflects stricter provisions in private standards in the UK, where for instance the Soil Association sets a limit at 2000 hens per hen house. Consequently, several hen houses per site are required to house larger numbers of hens.

According to the European regulation 889/2008 [18], a minimum of 4 m^2^ range area per hen is mandatory. In some countries, larger ranges are required: In the UK (Soil Association) as well as in AT (Codex alimentarius Austriacus) ≥10 m^2^ per hen are required and in DK ≥8 m^2^. The idea in DK is to provide 4 m^2^ range per hen in rotation and use it for poultry only every second year, but as long as there is sufficient vegetation on the range and the surface is at least doubled (≥8 m^2^), range rotation is not mandatory. The majority of the participating farms (70%) did not perform range rotation, although 39% of the farms had more than 8 m^2^ per hen available and consequently had the possibility to rotate the range area and at the same time always have ≥4 m^2^ accessible to the hens.

Weather conditions were the main reason for keeping hens inside. Snow was named most often as a reason to keep hens inside. Often hens are not believed to use the range if covered with snow, although the contrary was also observed during data collection for this study. However, concerns that the litter becomes moist and soiled may also play a role. Other common reasons for keeping the pop-holes closed were rain and low temperatures. Farmers are allowed to keep their hens inside in case of poor weather conditions as long as the hens have access to the free range for at least one third of their lifetime. In SE, the hens are kept indoors during the winter (November–March). Stadig et al. [50], from a survey among Belgian egg producers, also reported more free-range access in summer (11.1 ± 0.4 h/day) than in winter (7.0 ± 0.5 h/day).

In half of the countries (AT, BE, IT, UK), keeping cockerels with the hens was very uncommon (≤10% of farms), although previous studies showed a positive effect of cockerels on range use and the reduction of feather damage [13]. In SE, the proportion of flocks with cockerels was highest with 78%, but most kept less than 4 cockerels/1000 hens. The highest number of cockerels was 25/1000 in a German flock. The recommendation of some organic associations of 1 cock/100 hens (e.g., [51]), was only implemented in seven flocks in total. Beside the positive effects on free range use, there might be further positive effects of the presence of males on social behaviour and prevention of fear [52].

Free-range use during the two visits was relatively low, with on average 18.7% of hens outside (median: 13.6%); however, farms with closed pop-holes were included here. In comparison, Keppler et al. [53] reported an intensive range use around a frequently relocated mobile house of on average 35% of birds on the range at any given time over the day. Improved use of the outdoor area may be reached by providing shelters and other structuring elements, including bushes, hedges and trees [54,55]. Additionally, stocking density, group size, pop-hole design and light conditions in the hen house may influence the use of the free range [56,57,58].

The positive effect of range accessibility on plumage and skin conditions found in the current study [38] is in line with a number of other studies (reviewed by [10]). The importance of range access was further underlined by a negative association between worm burden (*A. galli*) and free-range access time [37], probably due to the worm eggs being spread over a much larger surface outdoors, thus reducing the likelihood of reinfection. Additionally, conditions for survival of the parasite eggs are less favourable on the range than inside. In conclusion, it is advisable to keep the pop-holes open for most of the day time and only close them if water provision would be endangered by the prevailing weather conditions (freezing), or if the thermoregulatory ability of the hens is impaired (e.g., by deteriorated plumage). Moreover, if the range is inaccessible, then other measures should be taken in order to offer the hens good quality environmental enrichment.

Mortality figures until 60 weeks (mean: 5.7%, median: 4.9%) were slightly lower in this study than reported elsewhere, e.g., from a Dutch study (mean until 60 weeks: 7.8%) [16]. They were also lower than the breeder’s guide reference value (8 to 10%) for Lohmann brown classic hens at 60 weeks of age under non-cage husbandry conditions [59]. However, the range of nearly 18 percentage points demonstrates large differences between flocks. As these numbers are based on farm records on daily losses and not on numbers of hens placed and slaughtered, they may not always reflect the complete record of lost hens (e.g., killed and removed by predators), but are based on hens found dead by hen keepers. The regression model explained 34% of the variance of mortality by three factors, namely laying performance at peak of lay, mite burden and use of the covered veranda. With the exception of the mite score, no association with the extent of specific health problems or the sum of different health problems in a flock could be detected. However, with each increase in mite score, mortality increased about 0.9% points. Sometimes, severe infestation with the poultry red mite (Dermanyssus gallinae) can cause death [60]. Yet, likely the major pathway was the infestation leading to general disturbance of the hens and impairment of their physical condition, e.g., weight loss and anaemia [61], thereby rendering them more susceptible to infections. In a more detailed statistical analysis of a small sample of the Danish data obtained in the current study, Hinrichsen et al. [62] found, in addition, that the mortality rate of flocks highly infected with helminths in the summer, but not in the winter, was increased two-fold compared to low-infection flocks. Concerning the endoparasite burden, they found, in more than half of the flocks (57.0%), more than 200 Ascarid eggs on average per gram faeces; this result was similar to earlier reports from Denmark, Sweden and Germany [21,63,64]. Mean ascarid EPG were positively correlated to *A. galli* worm burden in dissected hens, with mean worm burdens in 892 hens from 55 flocks of 10 *A. galli*/hen and 16 worms of *Heterakis spp.* with large variation between countries [37].

Regarding covered veranda use, lower mortality rates were observed where more hens had been counted. This may be due to the many potentially positive effects of a covered veranda: it helps to lower stocking densities inside the house, allows more effective withdrawal from unwanted contacts, provides good air quality combined with shelter, may promote free-range use and improve hygienic conditions and litter quality in the hen house by being an intermediate area to the free range.

The third significant factor in the model was hen-day egg production (%) at the peak of lay, with a negative association with mortality. The mean laying performances (in on average weeks 30 to 40) in our sample (90.6%) did not reach the genetic potential under optimum conditions of, e.g., Bovans white (95.2%) [65], nor did the performance reach that of Lohmann brown in non-cage systems (93.6%) [59] and had a considerable range of 26 percentage points, which, according to our results, reflected the general health condition of the flocks to a considerable degree. The mean laying performance of 81.4% towards the end of lay reflects an acceptable laying persistence, corresponding to the level expected, e.g., for Lohmann brown classic (84%) [59].

A higher average prevalence regarding foot pad lesions was found in the present study (30.5%) compared to an earlier study on Dutch organic hens (13%) [16]. Differences in scoring criteria may play a role. No significant model explaining the variation in foot pad lesion prevalences between farms in the present data could be constructed, although in the univariable selection of potential influencing factors, cleaner perches were significantly correlated with decreased prevalences of foot pad lesions (data not shown).

The prevalences of moderate (score 2) and severe (score 1) feather damage covered the whole range from 0 to 100% affected hens/flock in the present study, showing that there are farms without feather pecking problems, thereby indicating the potential for welfare improvement by appropriate management measures. Among them are adequate feeding, particularly regarding protein contents or ample access to the free range [38]. Skin lesions were generally less prevalent (92.5% of flocks had no lesions and a mean score of 3.8). Identified preventive measures were daily free-range access for brown hens and litter provision during the laying period in white hens [38].

The mean prevalence of keel bone damage (including deviations and palpable callus) of 44.5% was higher compared to former results from organic farms (28% and 21%) [6,66], but are comparable with prevalences reported from non-cage systems [67] and lower than reported by Heerkens et al. [25] for conventional and organic aviary systems in Belgium (fractures 82.5%, deviations 58.9%). Differences between study results may partly be due to differences in scoring criteria, e.g., different limits for the recording of callus material and deviations. In the current data set again variation between flocks was substantial. Four broad areas that could explain the high levels and variation of keel bone fractures independent of or in association with the level and persistence of laying performance have been identified by Toscano et al. [68]: the age at first egg, late ossification of the keel, predisposition to bone diseases and inactivity leading to poor bone health. Jung et al. [39], in the current data, identified aviary vs floor systems, absence of natural daylight in the hen house, a higher proportion of underweight birds as well as a higher laying performance as factors increasing the risk of keel bone damage. Donaldson et al. [69] showed that also poor house and aerial perches design are likely to be related to increased keel bone injuries. The increased prevalence in aviary systems calls for improved designs of these systems, including soft perches with better grip and as possible intermediate measures the provision of ramps [70,71,72].

## 5. Conclusions

HealthyHens is, to our knowledge, the largest and most detailed European study concerning animal welfare in organic laying hen farms. The information on some potential risk factors can be used for reducing health problems and mortality in European organic laying hen farms.

Efforts should be made in those countries with a low proportion of laying hens kept under organic husbandry conditions to ensure availability of organically raised pullets with intact beaks.

While production outcomes on the visited organic farms were on average comparable to breeder standards, results concerning the different welfare indicators showed room for improvement on many farms. The large ranges in the data set indicate at the same time that improvement is possible. According to our results, it is worthwhile to keep mite infestation and worm burden at low levels in order to prevent increased mortality. Hen-day egg production at peak of lay (in relation to breeder standards) appears to be a good indicator for the farmer of the health status of the flock. A central recommendation we can derive is to maximise access to the range, as this was related to lower infestation with *A. galli* as well as lower levels of feather damage and wounds. Additionally, an attractive covered veranda, which encourages the hens to make use of it, can reduce mortality. Further preventive measures, in particular against feather pecking and cannibalism, are sufficient protein levels in the feed and adequate litter provision. Good feeding is also important for the prevention of keel bone damage: sufficient weight of the birds (according to breeder standards) should be monitored and ensured. In the hen house, good light conditions and an adequate design must allow the birds to move between levels of the system without accidents. Thus, the results of the HealthyHens project point at a number of options for welfare improvement, both in terms of behavioural and health aspects, that are relatively easy to implement because they are primarily management-based measures. Based on this study and existing knowledge, practice-oriented recommendations have been developed and are available online (http://orgprints.org/20715/22/HealthyHensRecommendationLeaflet_english.pdf). We suggest that the results emphasise the need for increased knowledge transfer between science and farm practice.

## Figures and Tables

**Table 1:** Scores and definitions for the assessment of keel bone status, plumage status, skin lesions and foot pad lesions (in bold) in laying hens and achieved inter-assessor reliability (prevalence-adjusted bias-adjusted kappa values: PABAK).

Measure/Score			PABAK
**Keel Bone Deviation ^1^**			**0.8 (0.4–1)**
3	Straight or deviation < 0.5 cm		
2	Deviation > 0.5 cm to < 1 cm		
1	Deviation > 1 cm		
**Keel bone fracture ^1^**			0.7 (0.5–1)
2	No callus/pieces of fractured bone palpable		
1	Callus/pieces palpable		
**Plumage damage: neck, back, belly**		**Tail**	0.8 (0.4–1)
4	No or very few feathers damaged	≤5 tail feathers damaged	
3	Completely or almost completely feathered, few feathers damaged, featherless areas < 5 cm^2^	6–10 tail feathers damaged	
2	Highly damaged feathers and/or featherless areas. Featherless areas ≥ 5 cm^2^ (up to 75% featherless)	9–12 tail feathers highly damaged	
1	No or very few feather-covered areas. Featherless area ≥ 5 cm^2^ AND almost bare (75% featherless) up to completely featherless	≥13 tail feathers highly damaged and/or almost bare	
**Wounds on back and belly region**			Back 0.80 (0.4–1) Belly 0.65 (0.4–1)
4	No wounds at all		
3	Wounds < 0.5 cm or haematoma (no blood-filled follicles)		
2	Wounds ≥ 0.5 cm–2.2		
1	Wounds ≥ 2.2 cm		
**Foot pad lesions**			0.9 (0.8–1)
4	No lesion		
3	Small lesions < 0.2 cm		
2	Larger lesions > 0.2 cm		
1	Larger lesions ≥ 0.2 cm and dorsal swelling		

^1^ summed up to keel bone damage present or absent, keel bone tip (distal 1.5 cm of keel bone) not included.

**Table 2:** Criteria for the presence of health problems in a flock.

Indicator	Health Problem	Based on
Percentage of underweight hens (hens ≥ 11% points below breeder weight standards)	>10%	Keppler et al., (2016)
Plumage damage	<98% of hens with score 3 + 4	Jung and Knierim (2019)
Wounds	<100% of hens with score 4	Own definition
Percentage of hens with keel bone damage	>10%	Keppler et al. (2016)
Percentage of hens with foot pad lesions	>2%	Own definition
Mite score	≥3	Own definition
Ascaridia/Heterakis eggs per gram faeces	EPG > 200	Hinrichsen et al. (2016)
Coccidia oocysts per gram faeces	Mean EPG > 200	Own definition

**Table 3:** Distribution of dichotomous husbandry characteristics in a sample of 107 organic laying hen flocks in eight European countries.

Variables (Dichotomous)		Percentage of Flocks (Number)
Brown layers ^1^	Yes	78.5 (84)
	No	21.5 (23)
Beak trimmed	Yes	13.1 (14)
	No	86.9 (93)
Presence of cockerels ^2^	Yes	32.1 (34)
	No	67.9 (72)
Multi-tier system	Yes	35.5 (38)
	No	64.5 (69)
Disinfection of hen house after depopulation	Yes	85.9 (92)
	No	14.1 (15)
Range rotation ^3^	Yes	29.5 (31)
	No	70.5 (74)
Litter replacement or topping up during laying cycle	Yes	68.2 (73)
	No	31.8 (34)
Group nests	Yes	91.6 (98)
	No	8.4 (9)
Littered nests	Yes	24.3 (26)
	No	75.7 (81)
Dawn phase in light program	Yes	63.6 (68)
	No	36.4 (39)
Covered veranda	Yes	73.8 (79)
	No	26.2 (28)
Projecting roof ^4^	Yes	6.9 (7)
	No	93.1 (95)

^1^ Hens laying brown eggs; includes silver hybrids; ^2^ proportion ranging from 0.01 to 2.5%; ^3^ including temporary closing of (very) small parts of the range area in order to let the vegetation recover; ^4^ projecting at least 2 m; covered surface not closed/closable to all sides.

**Table 4:** Range (minimum, maximum), mean and median of continuous husbandry characteristics in a sample of 107 organic laying hen flocks assessed in eight European countries.

Variables (Continuous)	Min.	Max.	Mean	Median	N
Group size ^1^	318	4500	2422	2998	107
Total hen places of the farm (can be different sites)	550	305,000	13,762	6400	107
Hen house buildings per site	1	10	2.1	2	105
Cockerels per 1000 hens	0	25	1.9	0	106
Stocking density, hens/m^2^ accessible area indoors ^3^	2.4	7.5	5.2	5.3	102
Percent littered accessible area indoors	16	100	60.6	63	102
Days empty between batches	4	84	22.6	21	97
Access time (hours/day) to covered veranda	5	24	16.4	15	77
Clear height of pop-holes (cm) to free range as open at first visit	0	500	53.8	44	105
Dawn phase (min) in light schedule at first visit	0	90	16.5	15	107
Dusk phase (min) in light schedule at first visit	0	150	22.2	20	107

^1^ Groups were separated inside the building and in nearly all cases (four exceptions) also separated outside; ^3^ including raised surfaces without dunging pit/belt underneath and covered veranda if accessible 24 h/day.

**Table 5:** Range (minimum, maximum), means and medians for the production data and health outcomes around the peak and around the end of lay from organic laying hen flocks in eight European countries.

Variables (Continuous)	Min.	Max.	Mean	Median	N
Mortality until week 60 (%) ^1^	0.4	18.0	5.7	4.9	84
Mean hen-day egg production (%) at peak of lay (weeks 30 to 40) ^1^	72.9	98.6	90.6	92.1	88
Mean hen-day egg production (%) at end of lay (weeks 60 to 65) ^1^	60.7	97.0	81.4	83.7	84
% underweight hens	3.0	38.0	12.6	12.0	107
Mean score plumage damage	1.5	3.9	3.1	3.4	107
% flocks with >2% hens with score 1 + 2 (damaged plumage)			21.5		107
Mean score wounds at back or vent	2.0	4.0	3.8	3.9	107
% flocks with hens with wounds			7.5		107
% hens with damaged keel bones ^2^	2.9	88.0	44.5	44.0	107
% hens with foot pad lesions	0	80.0	30.5	29.0	106
Mite score at end of lay visit	0	5.0	1.8	2.0	71
% flocks with mite score ≥3			28.2		71
Mean *A. galli* and *Heterakis* eggs/g faeces (EPG) (mean of the two visits)	0	2450	449.4	284.9	103
% flocks with mean *A. galli* and *Heterakis* EPG >200			57.3		103
Mean Coccidia oocysts/g faeces (mean of the two visits)	0	9543	430.3	206.7	103
% flocks with mean coccidia oocysts/g faeces >200			57.3		103
% health problems per flock ^3^	12.5	100	62.3	62.5	107
% hens using the free range ^4^	0	79.6	18.7	13.6	107
% hens using the covered veranda ^4^	0	78.6	12.8	9.4	107
% hens using the covered veranda or the free range ^4^	0	93.9	31.5	29.0	107

^1^ week = week of life, based on farm records; ^2^ including deviations and fractures (assessed by palpation), excluding tip (about 1.5 cm); ^3^ number of health problems according to Table 2 in relation to all listed health problems in Table 2; ^4^ based on three scans each during two farm visits.

**Table 6:** Linear regression model for mortality rate in laying hens in % (n = 56).

Variables	Estimate	SE	t	*p*-Value
Intercept	35.285	7.020	5.026	<0.001
Hen-day egg production at peak of lay (%)	−0.334	0.078	−4.271	<0.001
Mite score	0.906	0.289	3.138	0.003
% hens using the covered veranda	−0.074	0.027	−2.723	0.009
